# Isolation, Characterization, and Compositional Analysis of Polysaccharides from Pinot Noir Wines: An Exploratory Study

**DOI:** 10.3390/molecules27238330

**Published:** 2022-11-29

**Authors:** Danye Zhu, Armando Alcazar-Magana, Yan Ping Qian, Yongsheng Tao, Michael C. Qian

**Affiliations:** 1 College of Enology, Northwest A&F University, Yangling 712100, China; 2 Department of Food Science and Technology, Oregon State University, Corvallis, OR 97331, USA; 3 Department of Crop and Soil Science, Oregon State University, Corvallis, OR 97331, USA; 4 Oregon Wine Research Institute, Oregon State University, Corvallis, OR 97331, USA

**Keywords:** wine polysaccharides, Pinot noir, sugar composition, size-exclusion chromatography, FT-IR, GC-MS

## Abstract

It has been reported that polysaccharides in wine can interact with tannins and other wine components and modify the sensory properties of the wine. Unfortunately, the contribution of polysaccharides to wine quality is poorly understood, mainly due to their complicated structure and varied composition. In addition, the composition and molecular structure of polysaccharides in different wines can vary greatly. In this study, the polysaccharides were isolated from pinot noir wine, then separated into high-molecular-weight (PNWP-H) and low-molecular-weight (PNWP-L) fractions using membrane-based ultrafiltration. Each polysaccharide fraction was further studied using size exclusion chromatography, UV–Vis, FT-IR, matrix-assisted laser desorption/ionization–high-resolution mass spectrometry, and gas chromatography-mass spectrometry (GC-MS). The results showed that PNWP-L and PNWP-H had different chemical properties and compositions. The FT-IR analysis showed that PNWPs were acidic polysaccharides with α- and β-type glycosidic linkages. PNWP-L and PNWP-H had different α- and β-type glycosidic linkage structures. FT-IR showed stronger antisymmetric and symmetric stretching vibrations of carboxylate anions of uronic acids in PNWP-L, suggesting more uronic acid in PNWP-L. The size exclusion chromatography results showed that over 72% of the PNWP-H fraction had molecular sizes from 25 kDa to 670 kDa. Only a small percentage of smaller molecular polysaccharides was found in the PNWP-H fraction. In comparison, all of the polysaccharides in the PNWP-L fraction were below 25 KDa, with a majority distributed approximately 6 kDa (95.1%). GC-MS sugar composition analysis showed that PNWP-L was mainly composed of galacturonic acid, rhamnose, galactose, and arabinose, while PNWP-H was mainly composed of mannose, arabinose, and galactose. The molecular size distribution and sugar composition analysis suggested that the PNWP-L primarily consisted of rhamnogalacturonans and polysaccharides rich in arabinose and galactose (PRAG). In comparison, PNWP-H were mostly mannoproteins and polysaccharides rich in arabinose and galactose (PRAG). Further research is needed to understand the impacts of these fractions on wine organoleptic properties.

## 1. Introduction

Many consumers much appreciate wine due to its organoleptic properties [1]. In addition to alcohol and tannins, polysaccharides comprise wine’s major class of macromolecules. The concentration of polysaccharides in wine varies greatly and can be as high as 2 g/L [2]. Wine polysaccharides originate from grape berries and yeast cells, so grape varieties and fermentation can affect their contents and compositions in the wine. The primary polysaccharides in wine are polysaccharides rich in arabinose and galactose (PRAG), rhamnogalacturonans (RG-I and RG-II), and mannoproteins (MP). PRAG comprises arabinogalactan proteins (AGP), arabinans, and arabinogalactans [2]. Although wine polysaccharides do not have a taste quality, they can interact with other wine components to affect the physical and organoleptic qualities of the wine [3,4]. For example, it has been reported that wine polysaccharides can stabilize wine colloids [5], improve wine foam and color stability [6,7], and inhibit tartaric crystal formation during wine storage [8]. In addition, wine polysaccharides can influence the aggregation of exogenous salivary protein–tannin complexes, affecting wine mouthfeel attributes and modulating wine astringency [9]. It has also been reported that wine polysaccharides can affect aroma perception [10,11,12]. The interactions are closely related to the structure and content of the polysaccharides [3].

Viticulture and enology practices can affect wine polysaccharide composition and characteristic profiles. It has been reported that grape maturity could influence the contents of PRAG, mannoproteins, and rhamnogalacturonans II in the wine [13,14]. Apolinar-Valiente et al. demonstrated that grape cultivars could affect the quantity and composition of the polysaccharides in wine [15]. Martínez Lapuente et al. found that longer pomace maceration time and high-power ultrasound treatment on crushed grapes significantly increased the content of PRAG and rhamnogalacturonans but not mannoprotein [16]. In other studies, it was observed that enzymatic treatments during the grape maceration stage led to a higher content of grape-derived pectic polysaccharides in the wine but not yeast-derived mannoproteins [17]. However, the pectic enzyme treatment modified the composition and structure of the polysaccharides, resulting in the loss of the terminal arabinose residues in PRAG [18]. The flash-release treatment of grapes, a wine-making practice consisting of rapidly heating the grapes and then applying a high vacuum to pull some volatiles, was also reported to enhance the extraction of PRAG and type II rhamnogalacturonan from the grapes [18].

The mannoproteins are derived from yeast during fermentation, whereas the arabinogalactan proteins and rhamnogalacturonans-II are derived from grapes. The distribution of the yeast-derived polysaccharides and grape-derived polysaccharides is dynamic during the maceration, fermentation, and aging processes. The grape-derived arabinogalactan proteins are the predominant polysaccharides in young wines, whereas the yeast-derived mannoproteins are the primary type in aged wines. Furthermore, the high molecular weight of mannoproteins and arabinogalactan proteins can precipitate during maturation, further complicating polysaccharide composition [19]. All aspects of the wine-making process affect the polysaccharides in the wine, so the “terroir effect” plays an important role in the polysaccharides composition [20].

Although significant progress has been made in recent years on wine polysaccharides, the chemical structures, composition, and properties of polysaccharides in different wines are largely unexplored. Wine polysaccharide characterization is a challenging field that needs to be explored to better understand the organoleptic functions of the wines. Hence, the scope of this study is to isolate polysaccharides from pinot noir wine and fractionate them based on molecular size using ultrafiltration, analyze their composition by measuring α- and ß-/furanosides and pyranosides derivative of each fractionation, and characterize each fraction using size-exclusion chromatography, UV–Vis, FT-IR, and matrix-assisted laser desorption/ionization–high-resolution mass spectrometry.

## 2. Results and Discussion

### 2.1. Pinot Noir Wine Polysaccharides Isolation and Fractionation

The polysaccharides (PNWPs) from pinot noir wine were concentrated using a membrane ultrafiltration technique. A 2 kDa membrane was successful in retaining the polysaccharides. A 100 kDa membrane could further fractionate the polysaccharides into low molecular weight (PNWP-L) and high molecular weight (PNWP-H) fractions. Next, ethanol was added to carefully precipitate the polysaccharides while leaving other impurities in the solution. The polysaccharides were first washed with 80% ethanol, followed by 100% ethanol until the supernatant was colorless. The process yielded 0.8 g PNWPs from 500 mL wines.

### 2.2. Yield and Impurity Analysis of PNWPs

The yields and impurity analysis of PNWPs are shown in Table 1. The yield of the PNWP-L and PNWP-H were 0.24 and 0.41 g/g PNWPs, respectively. The residual protein and total phenolics were analyzed. The proteins of the PNWP-H were higher than PNWP-L, which could be related to the presence of mannoprotein and arabinogalactan proteins in the PNWP-H. The total phenolic content of the PNWP-H was slightly higher than the PNWP-L, which could be related to the high molecular weight polyphenol aggregate in the PNWP-H fraction.

### 2.3. Ultraviolet-Visible Spectroscopy Analysis of PNWPs

**Figure 1:**
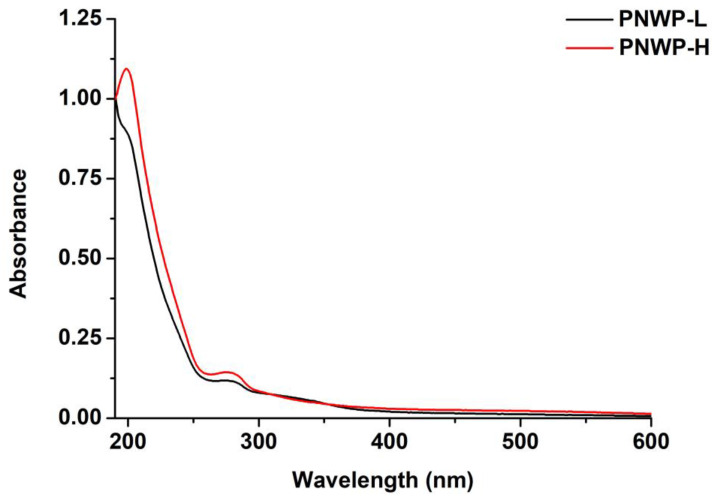
The UV–Vis spectra of PNWPs.

The UV-Vis spectra of PNWPs are shown in Figure 1. As illustrated in Figure 1, the isolated polysaccharides had minimal UV–Vis absorption from 250 to 600 nm except when the wavelength approached 200 nm. In addition, there was a small characteristic absorption peak at 260–280 nm, indicating a small amount of proteins and phenolics in PNWPs [21]. The presence of protein may be related to mannoproteins and arabinogalactan proteins (AGP) [22].

### 2.4. Fourier-Transform Infrared Spectroscopy Analysis of PNWPs

**Figure 2:**
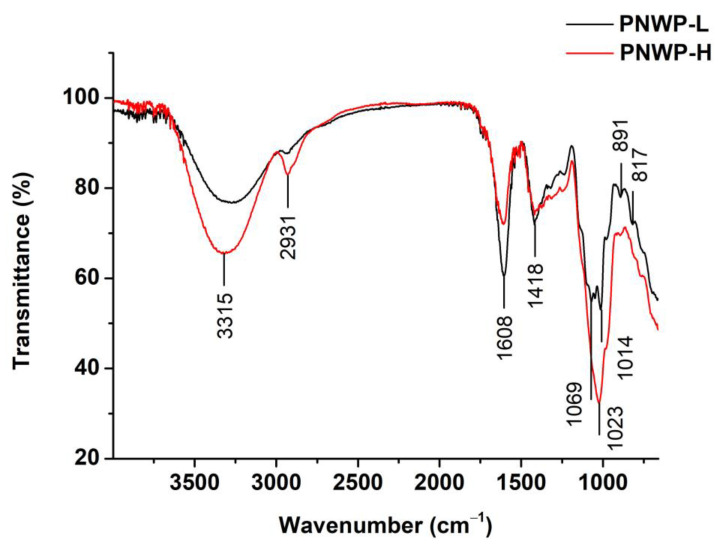
The FT-IR spectra of PNWPs.

FT-IR is an essential analytical technique for carbohydrate analysis, allowing for the rapid and non-destructive evaluation of structural information of wine polysaccharides [23]. The characteristic absorptions of the two polysaccharides with different molecular weights are identified in Figure 2. The broad peaks in the region of 3300–3500 cm^−1^ corresponded to the stretching vibration of the O-H, and the small bands near 2931 cm^−1^ represented the asymmetric vibration of C-H of polysaccharides [24]. The small peak at approximately 1748 cm^−1^ was derived from the C=O stretching vibration of the carbonyl group of the esters [25]. Two intense bands at 1608 cm^−1^ and 1418 cm^−1^ were associated with the antisymmetric and symmetric stretching vibrations of carboxylate anions (COO-) of uronic acids [23,26,27], and they were more pronounced for the PNWP-L, suggesting more uronic acid in the PNWP-L. Interestingly, the PNWP-H had much stronger absorption from 800 cm^−1^ to 1200 cm^−1^ than the PNWP-L. This wavelength region was associated with the stretching vibration of C-O-H (ring vibrations) and C-O-C (glycosidic bond), indicating different types of pyranose/furanose rings [28,29].

Moreover, the absorption at approximately 891 cm^−1^ could be attributed to the β-type glycosidic linkages [30], and the peak at approximately 817 cm^−1^ was likely related to α-type glycosidic linkages [31]. The FT-IR results showed that the PNWPs were acidic polysaccharides with α- and β-type glycosidic linkages, and the PNWP-L and the PNWP-H had different α- and β-type glycosidic linkage structures. The FT-IR spectra showed many shoulders on the major absorption. Boulet et al. reported that the numbers of peak shoulder of wine polysaccharides were as follows: AGP < MP < RG-II < RG-I. The number of peak shoulders of the PNWP-L was much more than the PNWP-H in the FT-IR spectrum, indicating that the PNWP-H contained more AGP and MP, while rhamnogalacturonans were the main fractions in the PNWP-L [22]. The absorption peaks in this region are unique and complex, but they are important to provide the fingerprint of molecules [32].

### 2.5. Molecular Weight Distribution of PNWPs

**Figure 3:**
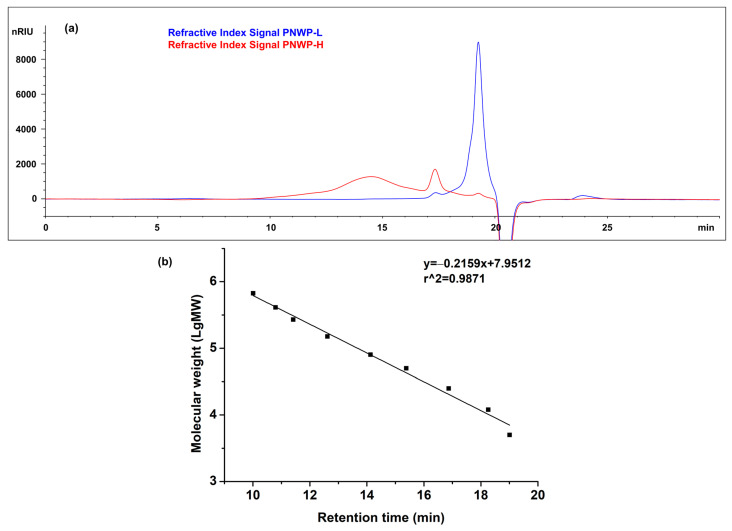
The molecular weight distribution chromatogram of PNWPs (**a**) and standard curve of dextrans plotted as retention time vs. Log MW (**b**).

Polysaccharide molecular sizes directly affect their physical properties. The molecular size of polysaccharides is typically measured by high-resolution size-exclusion chromatography with a refractive index detector using a standard curve [33] or multi-angle laser light scattering detectors [34]. The size-exclusion chromatograms of the PNWPs (a) and standard curve of dextrans (b) are presented in Figure 3. The molecular size distributions were calculated based on the peak area percentage (Table 2). The PNWP-H fraction had a wide large molecular size distribution from 25 kDa to 670 kDa, accounting for 72.3%. Only small percentages of smaller molecular size distributions were found in the PNWP-H fraction. In comparison, all of the PNWP-L fractions were below 25 KDa, with a majority of 6 kDa (95.1%). The results confirmed that the ultrafiltration technology used in this research effectively fractionated wine polysaccharides into low and high molecular weight fractions. The distribution of wine polysaccharide fractions can be related to many factors, such as grape variety, vintage, and wine-making techniques [3]. The molecular size distribution analysis showed that PNWPs were polysaccharides with a wide range of molecular sizes, consistent with the literature reports [16]. Jones-Moore et al. reported that the molecular weight of mannoproteins, arabinogalactan proteins, rhamnogalacturonans-II, and rhamnogalacturonans-I in wine was about 5–500 kDa, 50–260 kDa, 10 kDa, and 45–50 kDa, respectively [3]. Comparing the peak characteristics with that reported in the literature, it is likely that the peaks in the range of 9.5–17 min in PNWP-H mainly corresponded to the mannoproteins or mannans and the higher molecular weight of PRAG. The peak in the 17–18 min region mainly corresponded to the medium molecular weight of PRAG, mannoproteins or mannans, and rhamnogalacturonans. The peaks in the 18.5–20 min region mainly corresponded to rhamnogalacturonans, small fragments of PRAG, and mannoproteins or mannans [16,20,35]. Among them, PRAG and rhamnogalacturonans were derived from pinot noir grapes, while the mannoproteins and mannans were derived from yeast.

### 2.6. Matrix-Assisted Laser Desorption/Ionization-Time of Flight Mass Spectrometry (MALDI-TOF) Analysis of PNWP-L

**Figure 4:**
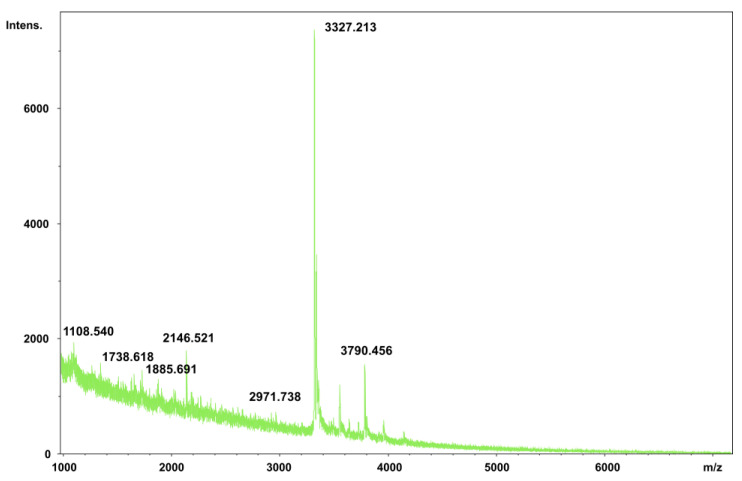
Matrix-assisted laser desorption/ionization-time of flight mass spectrum of the PNWP-L.

Multiple molecular weight determination methods were carried out in this study. In addition to the traditional SEC, MALDI-TOF analysis was also investigated. Compared to traditional SEC, MALDI-TOF analysis is a more advanced and accurate technique to determine the molecular weight of biological macromolecules, including low molecular weight polysaccharides. The MALDI-TOF spectrum of the PNWP-L is shown in Figure 4. A cluster of peaks is shown in the mass spectrum with the range of 1.11–3.79 kDa. Among them, the peak with a molecular weight of 3.33 kDa was the most prominent and was considered the main molecular weight of the PNWP-L. The large MW of polysaccharides do not ionize well for MALDI-TOF analysis. Selective enzyme hydrolysis followed by MALDI-TOF analysis will help elucidate polysaccharide branching and composition. One of the disadvantages of using MALDI-TOf-MS to characterize polysaccharides is the inefficiency in ionizing this class of molecules, especially when compared with peptides and proteins containing nitrogen atoms. Nevertheless, MALDI can provide accurate mass information and help elucidate the molecule’s sequence when the fragmentation spectra are analyzed.

### 2.7. Monosaccharide Composition Analysis of the PNWPs

#### 2.7.1. The Methanolysis Parameter Optimization

Monosaccharide profiling is critical to understanding the structure of wine polysaccharides. Typically, the wines are concentrated, and the small molecular weight compounds in the samples are removed by dialysis [4,16,34,36,37,38]. Next, the polysaccharides are precipitated using 80% ethanol and hydrolyzed with trifluoroacetic acid (TFA). The monosaccharide composition can then be analyzed by high-performance liquid chromatography [39]. Sugar composition can also be measured using GC or GC-MS by analyzing their trimethylsilyl-ester O-methyl glycosyl-derived (TMS) obtained after acidic methanolysis and derivatization [16,40], or their alditol acetate derivatives obtained after trifluoroacetic acid hydrolysis and derivatization [18]. GC and GC-MS techniques have better resolutions than the HPLC technique and are preferred for complex polysaccharide composition analysis. However, the methanolysis and derivatization conditions must be optimized for the polysaccharides to ensure complete methanolysis but without thermal artifact formation.

**Figure 5:**
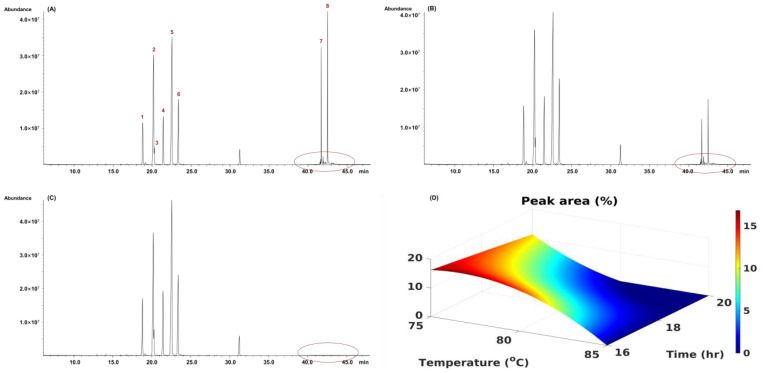
The methanolysis parameter optimization analysis of lactose. (**A**) 75 °C/18 h for methanolysis condition, 1. α-methyl 2,3,5,6-tetrakis-*O*-(trimethylsilyl)-galactofuranoside, 2. α-methyl 2,3,4,6-tetrakis-*O*-(trimethylsilyl)-galactopyranoside, 3. ß-methyl 2,3,5,6-tetrakis-*O*-(trimethylsilyl)-galactofuranoside, 4. ß-methyl 2,3,4,6-tetrakis-*O*-(trimethylsilyl)-galactopyranoside, 5. α-methyl 2,3,4,6-tetrakis-*O*-(trimethylsilyl)-glucopyranoside, and 6. ß-methyl 2,3,4,6-tetrakis-*O*-(trimethylsilyl)-glucopyranoside, 7. α-octakis(trimethylsilyl)-lactose, 8. ß-octakis(trimethylsilyl)-lactose; (**B**) 80 °C/18 h for methanolysis condition; (**C**) 85 °C/18 h for methanolysis condition; (**D**) the surface plot of the peak areas (%) of unmethanolyzed lactose and methanolysis conditions.

Lactose was selected for the methanolysis optimization study. The effect of temperature (75 °C, 80 °C, and 85 °C) and time (16 h, 18 h, and 20 h) on methanolysis for lactose were studied. Representative chromatograms under different methanolysis conditions are shown in Figure 5. In Figure 5A–C, the compounds with peak numbers 1–6 were α-methyl 2,3,5,6-tetrakis-*O*-(trimethylsilyl)-galactofuranoside, α-methyl 2,3,4,6-tetrakis-*O*-(trimethylsilyl)-galactopyranoside, ß-methyl 2,3,5,6-tetrakis-*O*-(trimethylsilyl)-galactofuranoside, ß-methyl 2,3,4,6-tetrakis-*O*-(trimethylsilyl)-galactopyranoside, α-methyl 2,3,4,6-tetrakis-*O*-(trimethylsilyl)-glucopyranoside, and ß-methyl 2,3,4,6-tetrakis-*O*-(trimethylsilyl)-glucopyranoside, respectively. The two distinct peaks (peak numbers 7–8) shown at 40–45 min were lactose-TMSs when the methanolysis conditions were carried out at lower temperatures or shorter times.

**Figure 6:**
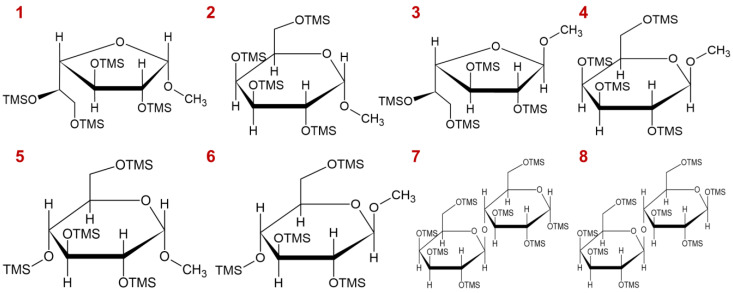
The structure of TMS derivatives corresponded to peaks 1–8 in Figure 5A.

As illustrated in Figure 5A–C, the lactose peaks decreased as the temperature increased from 75 °C to 85 °C. Therefore, the surface optimization experiment was conducted to determine the best conditions. Figure 5D shows the relationship between the peak area (%) of un-methanolyzed lactose and the methanolysis temperature and time. Based on the surface optimization chart, the optimal methanolysis condition was determined to be 85 °C for 18 h, and the conditions were used for subsequent composition analysis. The structures of TMS derivatives of the sugar are shown in Figure 6.

#### 2.7.2. Monosaccharide Composition of Pinot Noir Wine Polysaccharides

**Figure 7:**
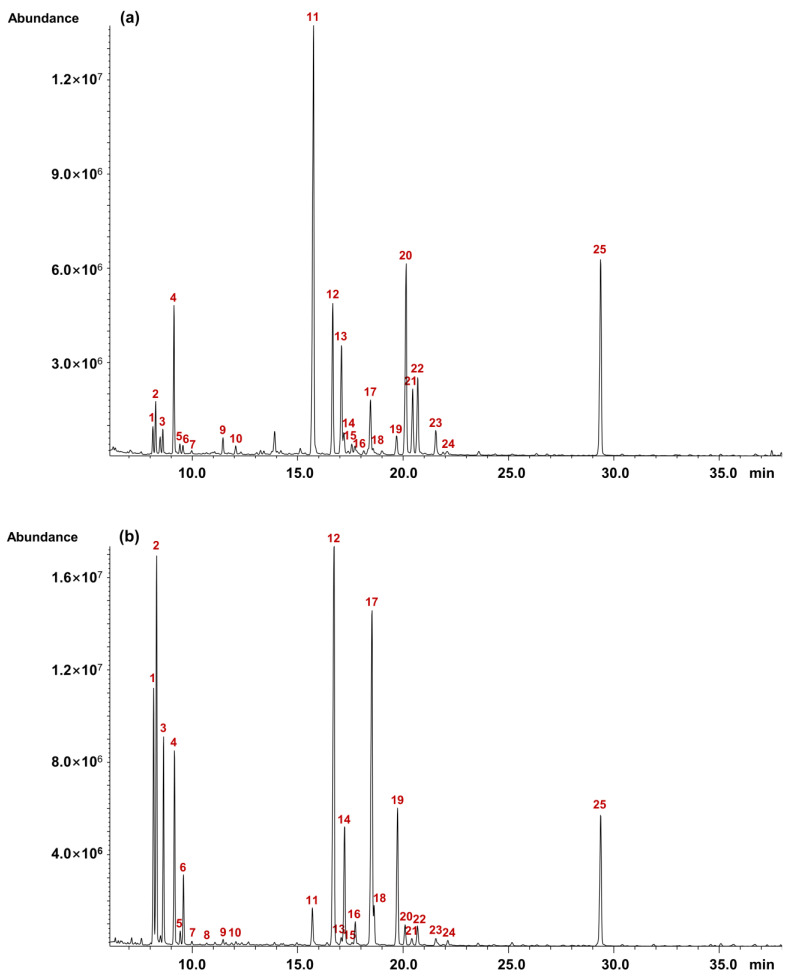
GC-MS chromatograms of PNWP-L (**a**) and PNWP-H (**b**). Peaks: 1. α-methyl 2,3,5-tris-*O*-(trimethylsilyl)-arabinofuranoside, 2. α-methyl 2,3,4-tris-*O*-(trimethylsilyl)-arabinopyranoside, 3. ß-methyl 2,3,4-tris-*O*-(trimethylsilyl)-arabinopyranoside, 4. α-methyl 2,3,4,6-tetrakis-*O*-(trimethylsilyl)-rhamnopyranoside, 5. ß-methyl 2,3,4,6-tetrakis-*O*-(trimethylsilyl)-rhamnopyranoside, 6. ß-methyl 2,3,5-tris-*O*-(trimethylsilyl)-arabinofuranoside, 7. α-methyl 2,3,4,6-tetrakis-*O*-(trimethylsilyl)-fucopyranoside, 8. ß-methyl 2,3,4,6-tetrakis-*O*-(trimethylsilyl)-fucopyranoside, 9. α-methyl 2,3,4-tris-*O*-(trimethylsilyl)-xylopyranoside, 10. ß-methyl 2,3,4-tris-*O*-(trimethylsilyl)-xylopyranoside, 11. α-methyl 2,3,5-tris-*O*-(trimethylsilyl)-galactofuranosiduronic acid, 12. α-methyl 2,3,4,6-tetrakis-*O*-(trimethylsilyl)-mannopyranoside, 13. ß-methyl 2,3,5-tris-*O*-(trimethylsilyl)-galactofuranosiduronic acid, 14. α-methyl 2,3,5,6-tetrakis-*O*-(trimethylsilyl)-galactofuranoside/α-methyl 2,3,5,6-tetrakis-*O*-(trimethylsilyl)-glucofuranoside, 15. ß-methyl 2,3,5,6-tetrakis-*O*-(trimethylsilyl)-glucofuranoside, 16. ß-methyl 2,3,4,6-tetrakis-*O*-(trimethylsilyl)-mannopyranoside, 17. α-methyl 2,3,4,6-tetrakis-*O*-(trimethylsilyl)-galactopyranoside, 18. ß-methyl 2,3,5,6-tetrakis-*O*-(trimethylsilyl)-galactofuranoside, 19. ß-methyl 2,3,4,6-tetrakis-*O*-(trimethylsilyl)-galactopyranoside, 20. α-methyl 2,3,4-tris-*O*-(trimethylsilyl)-galactopyranosiduronic acid, 21. ß-methyl 2,3,4-tris-*O*-(trimethylsilyl)-galactopyranosiduronic acid, 22. α-methyl 2,3,4,6-tetrakis-*O*-(trimethylsilyl)-glucopyranoside, 23. ß-methyl 2,3,4,6-tetrakis-*O*-(trimethylsilyl)-glucopyranoside/α-methyl 2,3,4-tris-*O*-(trimethylsilyl)-glucopyranosiduronic acid, 24. ß-methyl 2,3,4-tris-*O*-(trimethylsilyl)-glucopyranosiduronic acid, 25. myo-Inositol (hexakis-*O*-TMS).

The GC-MS chromatograms of PNWPs clearly illustrated the compositional differences between the PNWP-H and PNWP-L (Figure 7). As shown in Table 3, the PNWP-L and the PNWP-H exhibited significant differences in monosaccharide composition. Overall, the PNWP-L was mainly composed of α-galactofuranosiduronic acid (31.8%), α-galactopyranosiduronic acid (13.8%), α-mannopyranoside (9.9%), α-rhamnopyranoside (7.6%), ß-galactofuranosiduronic acid (7.3%), α-glucopyranoside (5.7%) and ß-galactopyranosiduronic acid (4.5%). By comparison, the PNWP-H was mainly composed of α-mannopyranoside (21.6%), α-galactopyranoside (18.7%), α-arabinopyranoside (13.1%), α-arabinofuranoside (8.4%), ß-galactopyranoside (6.9%), α-rhamnopyranoside (6.6%) and ß-arabinopyranoside (6.5%). Moreover, the contents of α-galactofuranosiduronic acid, ß-galactofuranosiduronic acid, α-galactopyranosiduronic acid, and ß-galactopyranosiduronic acid in the PNWP-L were much higher than the PNWP-H, which was consistent with FT-IR results. On the other hand, the contents of α-arabinofuranoside, ß-arabinopyranoside, α-arabinopyranoside, α-mannopyranoside, α-galactopyranoside, and ß-galactopyranoside in the PNWP-H were much higher than the PNWP-L. In addition, more uronic acid was detected in the PNWP-L compared to the PNWP-H. The above comparisons had significant differences based on significance analysis.

The Ara/Gal ratio was the feature of PRAG-related structures, and the value was related to the pectin hairy regions, and a higher value indicated a more pectin-related structure. The P-L had a total Ara content of 5.4%, and a total Gal content of approximately 7.0%. In comparison, the PNWP-H had a total Ara content of 30.4% and a total Gal content of approximately 33.0%. The Ara/Gal ratio for the PNWP-H was bigger than that of the PNWP-L, suggesting the PNWP-H had a more pectin-related structure. The Rha/GalA ratio was related to rhamnogalacturonan and homogalacturonans-related structures [41]. This ratio was about 1.8 for PNWH-H but only 0.1 for PNWH-L. Moreover, the (Ara + Gal)/Rha ratio was associated with the relative portion of the neutral side chains to the rhamnogalacturonans backbone [41]. The calculated (Ara + Gal)/Rha ratio of the PNWP-H was bigger than that of the PNWP-L. A higher value suggested more branch chains in the molecule. Vidal et al. reported that galacturonic acid and rhamnose were the main compositions in rhamnogalacturonans isolated from red wine [42]. In contrast, galactose and arabinose were the main components in arabinogalactan proteins, and mannose had the highest level of mannoproteins [42]. Watrelot et al. found that the main monosaccharides in pinot noir wines were galactose, mannose, and galacturonic acid, followed by glucose, arabinose, and a minimal amount of rhamnose, glucuronic acid and xylose [37]. In contrast, we found a relatively high percentage of arabinose in the PNWP-H fraction. Our result does not contradict the literature. It is anticipated that polysaccharide composition will depend on wine terroir, and different polysaccharide fractions will have different sugar compositions.

A higher percentage of galacturonic acid, rhamnose, galactose, and arabinose was found in the PNWP-L, suggesting the fraction mainly consisted of rhamnogalacturonans and PRAG. While the PNWP-H mainly contained mannose, arabinose, and galactose, suggesting it primarily consisted of mannoproteins and PRAG. The results demonstrated the polysaccharide compositions in wine were diverse and were derived from both grapes and wine. More research is needed to understand how the wine polysaccharide composition is related to grape varieties, enology practices, and maturation.

The complete elucidation of the wine polysaccharide structure is beyond the scope of this exploratory study. Detailed NMR experiments and branching analysis are underway to obtain more structural information and molecular characteristics.

## 3. Materials and Methods

### 3.1. Materials and Reagents

Pinot noir wines produced at Oregon State University Research Winery were used for wine polysaccharide isolation. All chemicals were of analytical grade unless otherwise specified. Ethanol (200 proof, HPLC-UV grade) was purchased from Pharmco (Brookfield, CT, USA). Acetyl chloride (≥99%), D-glucose (99%), D-galacturonic acid monohydrate (97%), L-arabinose (99%), L-fucose (99%), and L-rhamnose (99%) were purchased from Alfa Aesar (Tewksbury, MA, USA). Dextran standards, myo-inositol (≥99%), lactose (≥99%), and D-galactose (≥99%) were purchased from Sigma-Aldrich (Saint Louis, MO, USA). N-trimethylsilylimidazole (>98%) was bought from TCI Chemicals (Tokyo, Japan), ammonium formate (99%) was obtained from BeanTown Chemical (Hudson, NH, USA), and D-glucuronic acid (≥98%) was purchased from ICN Biomedicals (Irvine, CA, USA). Methanol (extra dry, 99.8%), pyridine (extra dry,99.5%), D-mannose (≥99%), and D-xylose (≥99%) were obtained from Acros Organics (Geel, Belgium).

### 3.2. PNWPs Extraction

**Figure 8:**
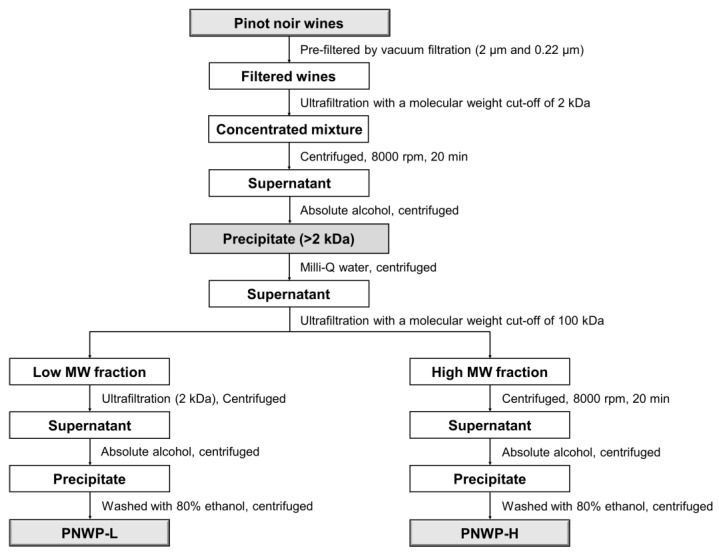
The extraction flow chat of PNWPs.

PNWPs were extracted using a previously reported method with minor modifications (Figure 8) [36,43,44]. Pinot noir wines were pre-filtered with a 2 µm glass fiber and 0.22 µm mixed cellulose esters membranes (Millipore Sigma, Burlington, MA, USA) by vacuum filtration. Subsequently, the filtered wine was subjected to ultrafiltration with a molecular weight cut-off of 2 kDa (Vivaflow 200, Sartorius, Göttingen, Germany) to remove small molecules such as residual sugars and organic acids. A volume of 500 mL of pinot noir wine was ultrafiltrated until reaching 30 mL. Then a small amount of Milli-Q water was added to continue the ultrafiltration process. The procedure was repeated a few more times to remove all small molecules. After ultrafiltration, the supernatant was collected and centrifuged at 8000 rpm for 20 min (Sorvall LYNX 4000 Centrifuge, Thermo Scientific, Waltham, MA, USA). Four volumes of absolute alcohol were added to the solution to make the ethanol concentration reach 80%, and the mixture was left at 4 °C overnight. The precipitates (above 2 kD) were harvested by centrifugation. The isolated polysaccharides were then redissolved in Milli-Q water and centrifuged again. The obtained supernatant was used for further fractionation with a molecular weight cut-off of 100 kDa ultrafiltration membrane. Two fractions of wine polysaccharides were collected, the PNWP-L for low molecular weight fraction from the filtrate, and the PNWP-H for high molecular weight fraction from the retentant. Both the PNWP-H and the PNWP-L were purified by ultrafiltration with a molecular weight cut-off of 2 kDa again. After precipitated with 80% ethanol, the precipitates were washed with 80% ethanol, followed by absolute ethanol until the supernatant was colorless. The samples were then centrifuged and filtered to recover the precipitates. The precipitates were dried in a vacuum oven (Forma Scientific, Waltham, MA, USA, with a high vacuum pump by Edwards, Crawley, UK) at 40 °C overnight. About 0.8 g polysaccharides with a molecular weight above 2 kD were obtained from 500 mL of wine. The isolation process was repeated 12 times to harvest sufficient quantities of polysaccharides for further study.

### 3.3. Protein and Total Phenol Analysis

Protein content was measured by the Bradford method with bovine serum albumin as a reference material [45]. The total phenolic content measurement was analyzed by the Folin–Ciocalteu colorimetric method using gallic acid as a standard, with some modifications [46].

### 3.4. Ultraviolet-Visible Spectroscopy Analysis

PNWPs were dissolved in Milli-Q water to a suitable concentration (0.1 mg/mL) and filled in a quartz cuvette. The ultraviolet spectrum of the PNWPs was scanned from 190 to 600 nm using a UV–visible spectrophotometer (UV-1800, Shimadzu, Japan). Water was used as blank according to the method described previously with minor modifications [47].

### 3.5. Fourier Transform Infrared Spectroscopy Analysis

The IR spectra of the PNWPs were obtained using an FT-IR spectrophotometer (Nicolet iS10, Thermo Scientific, Waltham, MA, USA) coupled with an Attenuated Total Reflectance sampling accessory (Smart iTR with diamond plate) with minor modifications [48]. The FTIR-ATR spectrum was recorded with a resolution of 4 cm^−1^ and 16 scans per spectrum in the 4000–600 cm^−1^ infrared region at room temperature. The background spectrum was collected in the air before each sample was measured. The sampling stage was cleaned with isopropanol after each measurement to avoid cross-contamination. The prominent absorption peaks of obtained results were performed using OMNIC 32 software and plotted as transmittance (%) vs. wave numbers (cm^−1^).

### 3.6. Molecular Weight Analysis

Molecular weight distribution was measured by high-performance size-exclusion chromatography with some modifications [48,49]. A Superose™ 6 Increase 10/300 (10 mm × 300 mm) GL column (Cytiva, Uppsala, Sweden) and an Agilent 1100 series HPLC system equipped with a refractive index detector (Agilent Technologies, Inc., Santa Clara, CA, USA) operated in positive polarity were used for separation and detection. The chromatographic conditions were as follows: mobile phase, 50 mM ammonium formate; column temperature, 35 °C; flow rate, 1 mL/min; injection volume, 10 μL. Dextrans with various molecular weights (5, 12, 25, 50, 80, 150, 270, 410, and 670 KDa) were used to create the calibration equations according to the elution time plotted against the logarithm of molecular weight.

### 3.7. Matrix-Assisted Laser Desorption Ionization-Time of Flight Mass Spectrometry (MALDI-TOF) Analysis

A small amount (1 mg) of the PNWP-L was mixed with 10 μL of 2,5-dihydroxybenzoic acid (DHB) matrix (50 mg/mL of DHB in 50% methanol and 0.1% TFA). The spectrum was recorded in an Ultraflex (Bruker Daltonics, Bremen, Germany) using negative and positive ion modes. Raw data were processed using DataAnalysis V4.1 (Bruker Daltonics).

### 3.8. Monosaccharide Composition Analysis

The methanlysis and derivatization optimization and gas chromatography-mass spectrometry (GC-MS) were performed according to previous methods with some modifications [50,51,52]. Lactose was selected for the methanolysis parameters optimization. The methanolysis temperature and time were investigated under anhydrous conditions for residual lactose after the reaction. The methanolysis conditions were evaluated at 75 °C, 80 °C, and 85 °C for 16 h, 18 h, and 20 h using a surface optimization design. Subsequently, the methanolysis, silylation, and GC-MS analysis were sequentially performed. The lowest reaction temperature and time combination was selected for complete methanolysis and minimum thermal artifact formation.

The monosaccharide composition of the PNWPs was determined by gas chromatography-mass spectrometry (GC-MS) using the optimized methanolysis and derivatization parameters. For the sugar composition analysis, about 0.7 mg of the PNWPs were dissolved in 0.5 mL 0.5 M dry HCl-methanol (acetyl chloride in dried methanol), and the methanolysis was performed at 85 °C for 18 h. After cooling to room temperature, the solution was mixed with 0.2 mL internal standard solution (0.1 mg/mL of myo-inositol in pyridine). The mixed solution was evaporated to dryness under a nitrogen gas flow. Finally, the residue was mixed with 0.1 mL N-trimethylsilylimidazole (TMSI) and incubated at 80 °C for 30 min. The final solution was analyzed by GC-MS on an Agilent 6890N-5973 system (Agilent Technologies, Inc., Santa Clara, CA, USA) equipped with an HP-5MS column (30 m × 0.25 mm × 0.25 μm, Agilent Technologies). The GC-MS program conditions were as follows: inlet temperature, 270 °C; hydrogen flow, 1.5 mL/min; split ratio: 10:1; the oven program (130 °C for 2 min; with 2 °C/min to 200 °C; with 20 °C/min to 280 °C, hold for 7 min). MS transfer line and ion source temperatures were 280 and 230 °C, respectively. Electron ionization mass spectrometric data from m/z 50 to 350 were collected with an ionization voltage of 70 eV.

The hybrid standard monosaccharides (glucose, xylose, mannose, arabinose, galactose, rhamnose, fucose, glucuronic acid, and galacturonic acid) were derivatized and analyzed under the same conditions described previously. Identification was achieved with the standard compounds and with the NIST library (Rev. D05.01). The calculation was processed using ChemStation software (ver. E.02, Agilent Technologies Inc., Santa Clara, CA, USA).

### 3.9. Statistical Analysis

The results were exhibited as mean ± standard deviation (SD) in triplicate (n = 3). Duncan’s test and an independent sample *t*-test were performed by SPSS statistics 20 software; *p* < 0.05 was considered significant.

## 4. Conclusions

In this study, two fractions of wine polysaccharides were isolated by ultrafiltration technology. FT-IR results showed that the PNWPs were acidic polysaccharides with α- and β-type glycosidic linkages. GC-MS analysis revealed the PNWP-L was mainly composed of α-galactofuranosiduronic acid, α-galactopyranosiduronic acid, α-mannopyranoside, α-rhamnopyranoside, ß-galactofuranosiduronic acid, α-glucopyranoside, and ß-galactopyranosiduronic acid. In comparison, the PNWP-H was mainly composed of α-mannopyranoside, α-galactopyranoside, α-arabinopyranoside, α-arabinofuranoside, ß-galactopyranoside, α-rhamnopyranoside, and ß-arabinopyranoside. Moreover, relatively more glucuronic acid in the PNWP-L was found. The compositional analysis and molecular weight characterization showed that the low molecular weight polysaccharides in pinot noir wine were mainly rhamnogalacturonans and polysaccharides rich in arabinose and galactose (PRAG). In contrast, the high molecular weight polysaccharides in pinot noir wine were mainly mannoproteins and polysaccharides rich in arabinose and galactose (PRAG). This information provided the molecular basis to further study the effect of these polysaccharides on the organoleptic attributes of pinot noir wine.

## Figures and Tables

**Table 1:** The yield and chemical component analysis of PNWPs.

Samples	Yield (g/g) ^#^	Protein Content (%)	Total Phenolic Content (%)
PNWP-L	0.24	0.3% ± 0.1%	1.7% ± 0.0%
PNWP-H	0.41	5.3% ± 0.4%	2.2% ± 0.0%

^#^—the fractions obtained from PNWPs.

**Table 2:** The molecular weight distribution (as dextran equivalent) and peak area percentage of PNWPs.

Samples	Peak No.	Peak MW (kDa)	MW Distribution (kDa)	Area Account (%)
PNWP-L	1	15.9 ± 0.0	12–25	4.9
	2	6.2 ± 0.0	5–12	95.1
PNWP-H	1	66.5 ± 0.3	25–670	72.3
	2	16.2 ± 0.0	12–25	22.1
	3	6.2 ± 0.0	5–12	5.6

**Table 3:** The monosaccharide composition of PNWPs.

Peak No.	Composition	Monosaccharide Composition Percentage (%)		*p* Value ^#^
		PNWP-L	PNWP-H	
1	α-Ara*f*	1.3 ± 0.0 l	8.4 ± 0.1 d	*
2	α-Ara*p*	2.4 ± 0.1 i	13.1 ± 0.2 c	*
3	ß-Ara*p*	1.2 ± 0.0 l	6.5 ± 0.1 f	*
4	α-Rha*p*	7.6 ± 0.2 d	6.6 ± 0.1 f	*
5	ß-Rha*p*	0.6 ± 0.0 no	0.5 ± 0.0 l	-
6	ß-Ara*f*	0.5 ± 0.0 o	2.4 ± 0.1 h	*
7	α-Fuc*p*	0.2 ± 0.0 p	0.1 ± 0.0 n	-
8	ß-Fuc*p*	-q	0.1 ± 0.0 n	*
9	α-Xyl*p*	1.0 ± 0.1 m	0.2 ± 0.0 mn	*
10	ß-Xyl*p*	0.5 ± 0.0 o	0.1 ± 0.0 n	*
11	α-Gal*f*A	31.8 ± 0.3 a	2.1 ± 0.3 i	*
12	α-Man*p*	9.9 ± 0.2 c	21.6 ± 0.4 a	*
13	ß-Gal*f*A	7.3 ± 0.1 e	0.4 ± 0.1 lm	*
14	α-Gal*f*/α-Glc*f*	1.5 ± 0.0 k	5.8 ± 0.2 g	*
15	ß-Glc*f*	0.7 ± 0.0 n	0.1 ± 0.0 n	*
16	ß-Man*p*	0.9 ± 0.0 m	1.2 ± 0.0 k	*
17	α-Gal*p*	4.3 ± 0.1 h	18.7 ± 0.2 b	*
18	ß-Gal*f*	0.5 ± 0.0 o	1.6 ± 0.2 j	*
19	ß-Gal*p*	1.5 ± 0.0 k	6.9 ± 0.1 e	*
20	α-Gal*p*A	13.8 ± 0.2 b	1.1 ± 0.1 k	*
21	ß-Gal*p*A	4.5 ± 0.0 g	0.4 ± 0.0 lm	*
22	α-Glc*p*	5.7 ± 0.0 f	1.0 ± 0.0 k	*
23	ß-Glc*p*/α-Glc*p*A	1.9 ± 0.0 j	0.5 ± 0.0 l	*
24	ß-Glc*p*A	0.3 ± 0.0 p	0.2 ± 0.0 mn	-

Abbreviation notes: *f*—furanoside type; *p*—pyranoside type. Ara—arabinose, Rha—rhamnose, Fuc—fucose, Xyl—xylose, GalA—galacturonic acid Man—mannose, Gal—galactose, Glc—glucose, GlcA—glucuronic acid. Lowercase letters indicate significant differences (*p* < 0.05) between the different monosaccharides in PNWPs. ^#^ *p*-value was performed by an independent sample *t*-test (*p* < 0.05), * means significant, - means not significant.

## Data Availability

The data presented in this study are available on request from the corresponding authors.

## Sample Availability

Samples are available from the corresponding authors upon request.

## References

[1] Perez-Jimenez M., Sherman E., Pozo-Bayon M.A., Pinu F.R. (2021). Application of untargeted volatile profiling and data driven approaches in wine flavoromics research. Food Res. Int..

[2] Jones-Moore H.R., Jelley R.E., Marangon M., Fedrizzi B. (2021). The polysaccharides of winemaking: From grape to wine. Trends Food Sci. Technol..

[3] Jones-Moore H.R., Jelley R.E., Marangon M., Fedrizzi B. (2022). The interactions of wine polysaccharides with aroma compounds, tannins, and proteins, and their importance to winemaking. Food Hydrocoll..

[4] Mitropoulou A., Hatzidimitriou E., Paraskevopoulou A. (2011). Aroma release of a model wine solution as influenced by the presence of non-volatile components. Effect of commercial tannin extracts, polysaccharides and artificial saliva. Food Res. Int..

[5] Garrido-Banuelos G., Buica A., Schuckel J., Zietsman A.J.J., Willats W.G.T., Moore J.P., Du Toit W.J. (2019). Investigating the relationship between grape cell wall polysaccharide composition and the extractability of phenolic compounds into Shiraz wines. Part I: Vintage and ripeness effects. Food Chem..

[6] Martinez-Lapuente L., Apolinar-Valiente R., Guadalupe Z., Ayestaran B., Perez-Magarino S., Williams P., Doco T. (2018). Polysaccharides, oligosaccharides and nitrogenous compounds change during the ageing of Tempranillo and Verdejo sparkling wines. J. Sci. Food Agric..

[7] Guadalupe Z., Palacios A., Ayestarán B. (2007). Maceration enzymes and mannoproteins: A possible strategy to increase colloidal stability and color extraction in red wines. J. Agric. Food Chem..

[8] Filipe-Ribeiro L., Milheiro J., Guise R., Vilamarim R., Fraga J.B., Martins-Gomes C., Nunes F.M., Cosme F. (2021). Efficiency of carboxymethylcellulose in red wine tartaric stability: Effect on wine phenolic composition, chromatic characteristics and colouring matter stability. Food Chem..

[9] Kassara S., Li S., Smith P., Blando F., Bindon K. (2019). Pectolytic enzyme reduces the concentration of colloidal particles in wine due to changes in polysaccharide structure and aggregation properties. Int. J. Biol. Macromol..

[10] Laguna L., Bartolomé B., Moreno-Arribas M.V. (2017). Mouthfeel perception of wine: Oral physiology, components and instrumental characterization. Trends Food Sci. Technol..

[11] Chong H.H., Cleary M.T., Dokoozlian N., Ford C.M., Fincher G.B. (2019). Soluble cell wall carbohydrates and their relationship with sensory attributes in Cabernet Sauvignon wine. Food Chem..

[12] Dufour C., Bayonove C.L. (1999). Influence of wine structurally different polysaccharides on the volatility of aroma substances in a model system. J. Agric. Food Chem..

[13] Gil M., Kontoudakis N., Gonzalez E., Esteruelas M., Fort F., Canals J.M., Zamora F. (2012). Influence of grape maturity and maceration length on color, polyphenolic composition, and polysaccharide content of Cabernet Sauvignon and Tempranillo wines. J. Agric. Food Chem..

[14] Martinez-Lapuente L., Apolinar-Valiente R., Guadalupe Z., Ayestaran B., Perez-Magarino S., Williams P., Doco T. (2016). Influence of Grape Maturity on Complex Carbohydrate Composition of Red Sparkling Wines. J. Agric. Food Chem..

[15] Apolinar-Valiente R., Romero-Cascales I., Williams P., Gómez-Plaza E., López-Roca J.M., Ros-García J.M., Doco T. (2014). Effect of winemaking techniques on polysaccharide composition of Cabernet Sauvignon, Syrah and Monastrell red wines. Aust. J. Grape Wine Res..

[16] Martinez Lapuente L., Guadalupe Z., Ayestaran B., Perez-Porras P., Bautista-Ortin A.B., Gomez-Plaza E. (2021). Ultrasound treatment of crushed grapes: Effect on the must and red wine polysaccharide composition. Food Chem..

[17] Ayestarán B., Guadalupe Z., León D. (2004). Quantification of major grape polysaccharides (*Tempranillo* v.) released by maceration enzymes during the fermentation process. Anal. Chim. Acta.

[18] Doco T., Williams P., Cheynier V. (2007). Effect of flash release and pectinolytic enzyme treatments on wine polysaccharide composition. J. Agric. Food Chem..

[19] Guadalupe Z., Ayestarán B. (2007). Polysaccharide profile and content during the vinification and aging of Tempranillo red wines. J. Agric. Food Chem..

[20] Apolinar-Valiente R., Williams P., Romero-Cascales I., Gomez-Plaza E., Lopez-Roca J.M., Ros-Garcia J.M., Doco T. (2013). Polysaccharide composition of Monastrell red wines from four different Spanish terroirs: Effect of wine-making techniques. J. Agric. Food Chem..

[21] Jeong H.K., Lee D., Kim H.P., Baek S.H. (2019). Structure analysis and antioxidant activities of an amylopectin-type polysaccharide isolated from dried fruits of *Terminalia chebula*. Carbohydr. Polym..

[22] Boulet J.C., Williams P., Doco T. (2007). A Fourier transform infrared spectroscopy study of wine polysaccharides. Carbohydr. Polym..

[23] Baca-Bocanegra B., Martinez-Lapuente L., Nogales-Bueno J., Hernandez-Hierro J.M., Ferrer-Gallego R. (2022). Feasibility study on the use of ATR-FTIR spectroscopy as a tool for the estimation of wine polysaccharides. Carbohydr. Polym..

[24] Gheribi R., Habibi Y., Khwaldia K. (2019). Prickly pear peels as a valuable resource of added-value polysaccharide: Study of structural, functional and film forming properties. Int. J. Biol. Macromol..

[25] Sharma M., Aguado R., Murtinho D., Valente A.J.M., Ferreira P.J.T. (2021). Novel approach on the synthesis of starch betainate by transesterification. Int. J. Biol. Macromol..

[26] Dong X., Zhu C.-P., Huang G.-Q., Xiao J.-X. (2021). Fractionation and structural characterization of polysaccharides derived from red grape pomace. Process Biochem..

[27] Kostalova Z., Hromadkova Z. (2019). Structural characterisation of polysaccharides from roasted hazelnut skins. Food Chem..

[28] Hong T., Yin J.Y., Nie S.P., Xie M.Y. (2021). Applications of infrared spectroscopy in polysaccharide structural analysis: Progress, challenge and perspective. Food Chem. X.

[29] Liu J., Liu C., Zheng X., Chen M., Tang K. (2020). Soluble soybean polysaccharide/nano zinc oxide antimicrobial nanocomposite films reinforced with microfibrillated cellulose. Int. J. Biol. Macromol..

[30] Zhang J., Wen C., Gu J., Ji C., Duan Y., Zhang H. (2019). Effects of subcritical water extraction microenvironment on the structure and biological activities of polysaccharides from Lentinus edodes. Int. J. Biol. Macromol..

[31] Yang B., Wu Q., Luo Y., Yang Q., Wei X., Kan J. (2019). High-pressure ultrasonic-assisted extraction of polysaccharides from Hovenia dulcis: Extraction, structure, antioxidant activity and hypoglycemic. Int. J. Biol. Macromol..

[32] Chen X., Jin J., Tang J., Wang Z., Wang J., Jin L., Lu J. (2011). Extraction, purification, characterization and hypoglycemic activity of a polysaccharide isolated from the root of *Ophiopogon japonicus*. Carbohydr. Polym..

[33] Del Barrio-Galán R., Pérez-Magariño S., Ortega-Heras M., Guadalupe Z., Ayestarán B. (2012). Polysaccharide characterization of commercial dry yeast preparations and their effect on white and red wine composition. LWT Food Sci. Technol..

[34] Cordeiro Caillot A.R., de Lacerda Bezerra I., Palhares L., Santana-Filho A.P., Chavante S.F., Sassaki G.L. (2018). Structural characterization of blackberry wine polysaccharides and immunomodulatory effects on LPS-activated RAW 264.7 macrophages. Food Chem..

[35] Ducasse M.-A., Canal-Llauberes R.-M., de Lumley M., Williams P., Souquet J.-M., Fulcrand H., Doco T., Cheynier V. (2010). Effect of macerating enzyme treatment on the polyphenol and polysaccharide composition of red wines. Food Chem..

[36] Lei X., Zhu Y., Wang X., Zhao P., Liu P., Zhang Q., Chen T., Yuan H., Guo Y. (2019). Wine polysaccharides modulating astringency through the interference on interaction of flavan-3-ols and BSA in model wine. Int. J. Biol. Macromol..

[37] Watrelot A.A., Schulz D.L., Kennedy J.A. (2017). Wine polysaccharides influence tannin-protein interactions. Food Hydrocoll..

[38] Carvalho E., Mateus N., Plet B., Pianet I., Dufourc E., De Freitas V. (2006). Influence of wine pectic polysaccharides on the interactions between condensed tannins and salivary proteins. J. Agric. Food Chem..

[39] Wan M., Wang M., Zhao Y., Deng H., Tan C., Lin S., Kong Y., Tong Y., Meng X. (2021). Extraction of mannoprotein from *Saccharomyces cerevisiae* and analysis of its chemical composition and molecular structure. Int. J. Biol. Macromol..

[40] Guadalupe Z., Martínez-Pinilla O., Garrido Á., Carrillo J.D., Ayestarán B. (2012). Quantitative determination of wine polysaccharides by gas chromatography–mass spectrometry (GC–MS) and size exclusion chromatography (SEC). Food Chem..

[41] Canalejo D., Guadalupe Z., Martinez-Lapuente L., Ayestaran B., Perez-Magarino S., Doco T. (2022). Characterization of polysaccharide extracts recovered from different grape and winemaking products. Food Res. Int..

[42] Vidal S., Williams P., Doco T., Moutounet M., Pellerin P. (2003). The polysaccharides of red wine: Total fractionation and characterization. Carbohydr. Polym..

[43] Eder S., Zueblin P., Diener M., Peydayesh M., Boulos S., Mezzenga R., Nystrom L. (2021). Effect of Polysaccharide Conformation on Ultrafiltration Separation Performance. Carbohydr. Polym..

[44] Lan H., Nunes C., Lopes G.R., Wang K., Zhao L., Coimbra M.A., Hu Z. (2021). In vitro immunomodulatory activity of water-soluble glucans from fresh and dried Longan (*Dimocarpus longan* Lour.). Carbohydr. Polym..

[45] Mohammed J.K., Mahdi A.A., Ahmed M.I., Ma M., Wang H. (2020). Preparation, deproteinization, characterization, and antioxidant activity of polysaccharide from *Medemia argun* fruit. Int. J. Biol. Macromol..

[46] Lin X., Ji X., Wang M., Yin S., Peng Q. (2019). An alkali-extracted polysaccharide from *Zizyphus jujuba* cv. Muzao: Structural characterizations and antioxidant activities. Int. J. Biol. Macromol..

[47] Wang L., Li X., Wang B. (2018). Synthesis, characterization and antioxidant activity of selenium modified polysaccharides from *Hohenbuehelia serotina*. Int. J. Biol. Macromol..

[48] Wei W., Li J., Qi X., Zhong Y., Zuo G., Pan X., Su T., Zhang J., Dong W. (2017). Synthesis and characterization of a multi-sensitive polysaccharide hydrogel for drug delivery. Carbohydr. Polym..

[49] Canalejo D., Guadalupe Z., Martinez-Lapuente L., Ayestaran B., Perez-Magarino S. (2021). Optimization of a method to extract polysaccharides from white grape pomace by-products. Food Chem..

[50] Sran K.S., Sundharam S.S., Krishnamurthi S., Roy Choudhury A. (2019). Production, characterization and bio-emulsifying activity of a novel thermostable exopolysaccharide produced by a marine strain of *Rhodobacter johrii* CDR-SL 7Cii. Int. J. Biol. Macromol..

[51] Liu D., Tang W., Yin J.-Y., Nie S.-P., Xie M.-Y. (2021). Monosaccharide composition analysis of polysaccharides from natural sources: Hydrolysis condition and detection method development. Food Hydrocoll..

[52] Wang Z., Zeng Y., Luo D. (2016). Structure elucidation of a non-branched and entangled heteropolysaccharide from *Tremella sanguinea* Peng and its antioxidant activity. Carbohydr. Polym..

